# Differences in spousal influence on smoking cessation by gender and education among Japanese couples

**DOI:** 10.1186/1471-2458-14-1184

**Published:** 2014-11-19

**Authors:** Daisuke Takagi, Naoki Kondo, Misato Takada, Hideki Hashimoto

**Affiliations:** Department of Health and Social Behavior, The University of Tokyo, 7-3-1 Hongo, Bunkyo-ku, Tokyo, 113-0033 Japan

**Keywords:** Japan, Smoking cessation, Spousal influence, Education

## Abstract

**Background:**

Previous studies have reported that spousal non-smoking has a spillover effect on the partner’s cessation. However, discussion is lacking on the factors modifying that association. We examined whether the spillover effect of spousal non-smoking was associated with the couple’s educational attainment.

**Methods:**

We used paired marital data from the Japanese Study on Stratification, Health, Income, and Neighborhood (J-SHINE), which targeted residents aged 25–50 years in four Japanese municipalities. We selected a spouse smoker at the time of marriage (target respondent), and set his/her smoking status change (continued or quit smoking after marriage) as an outcome, regressed on the counterpart’s smoking status (continued smoking or non-smoking) and combinations of each couple’s educational attainment as explanatory variables using log-binomial regression models (*n* =1001 targets; 708 men and 293 women).

**Results:**

Regression results showed that a counterpart who previously quit smoking or was a never-smoker was associated with the target male spouse’s subsequent cessation. However, for women, the association between husband’s non-smoking and their own cessation was significant only for couples in which both spouses were highly educated.

**Conclusions:**

Our findings suggest that a spouse’s smoking status is important for smoking cessation interventions in men. For women, however, a couple’s combined educational attainment may matter in the interventions.

## Background

Smoking is a risk factor associated with the incidence of various non-communicable diseases. Owing to secondhand smoke, smoking is harmful even to non-smokers, making it a major target of public health intervention, not only for smokers but also their families and neighbors.

In common with developed Western countries, the smoking rate in Japan has declined markedly since the 1950s. However, the male smoking rate in Japan is the sixth highest among OECD (Organization for Economic Cooperation and Development) countries [1], and tobacco remains the largest contributor to the nation’s burden of diseases [2]. There is a notable trend in the prevalence of smoking among women with respect to age: although the female smoking rate in Japan is relatively low compared with Western countries, the rate among young women is increasing [3].

Among individual characteristics affecting smoking initiation and cessation, researchers have focused on age [4], gender [5], educational background [6, 7], and other sociodemographic factors [8]. In parallel with the studies of individual factors, many researchers have also studied the influence of family, especially a spouse, on smoking cessation among married people [9–12]. Spousal smoking status is regarded as a significant factor affecting a partner’s smoking cessation. A longitudinal study by Falba and Sindelar [13] found that, if a spouse quit smoking, the odds of the partner’s cessation showed an increase of up to 7.5-fold among men and 8.5-fold among women. Using self-reported life histories of smoking behavior, Monden et al. [10] found that respondents living with an ex-smoker or never-smoker spouse were more likely to quit smoking than respondents living with a current smoker.

Despite a number of publications linking a spouse’s smoking cessation to that of their partner, discussion of the factors modifying these associations is lacking. As Ross et al. [14] discussed in their review paper, a couple’s key characteristic in affecting the concordance of health-related behaviors is educational background. Many studies suggested that an individual’s education affects their own health through material circumstances, behavioral factors (e.g., lifestyle), and psychosocial factors (e.g., social support) [14–16]. A spouse’s education may also affect their partner’s health by the same mechanisms as the individual’s own education [17]. Therefore, taking a spouse’s education into account may well explain the health of married people. For example, using cross-sectional data, Bloch et al. [18] found that highly educated couples showed a high concordance rate with respect to absence of smoking behavior. Monden et al. [17] also examined the effect of a participant’s own and their spouse’s educational attainment on smoking behavior using data from about 40,000 Dutch people. Although the results suggested that a spouse’s education was significantly associated with their partner’s smoking behavior, the effect was weaker than that of the participant’s own educational level. However, because these studies did not examine when the couples started and stopped smoking, they could not conclude whether the observed association was the result of a real *spillover effect* of the partner’s behavior change or just the reflection of *assortative mating.* Based on social exchange theory, assortative mating proposes that mate selection is not random and that individuals are likely to choose a partner who is similar in personality, behavior, physical features, and health [19–22].

To find a leverage point of behavioral intervention to reduce smoking, it would be beneficial for public health practitioners to know whether couples’ behavioral interactions and their educational backgrounds affect the likelihood of smoking cessation. If the spillover effect exists and the effect is influenced by the couple’s educational backgrounds, we can more effectively modify smoking cessation interventions according to their own and their partners’ educational statuses whereas if the assortative mating explanation is a dominant factor for the couple’s concordance in smoking behavior, approaches relevant to the target’s educational background would not work. Thus, this paper examined how an individual’s smoking cessation is affected by a spouse’s prior smoking behavior status, and how these associations are altered by the couple’s educational attainment combinations.

## Methods

### Data

Data from the Japanese Study on Stratification, Health, Income, and Neighborhood (J-SHINE) project were used for this study, details of which are described elsewhere [23]. The wave 1 survey was conducted from July 2010 to February 2011 in four municipalities in Japan (two in the Tokyo metropolitan area and two in a nearby prefecture), with a probabilistic sample of community-dwelling men and women aged 25–50 years. The sample size of the wave 1 survey was 8,408, and 4,357 respondents replied (response rate was 51.8%). Among them, 3,027 with a spouse or partner were invited to take part in a spouse/partner survey from August to December 2011; this involved asking similar questions as in the wave 1 survey questionnaire to make a pair-wise comparison. The questionnaire was filled out by the spouses themselves. Data from the spouse/partner survey were merged into the wave 1 data, and the paired data of 1,500 couples were available for the analysis. All couples were asked when they married, and when each member of the couple initiated/quit smoking. Thirty-two couples in which spouses were living together but were not legally married were not included in our analyses because there was no question identifying the starting date of their partner relationship. Additionally, 545 couples in which both spouses were non-smokers at the time of marriage were also omitted from our analyses. The present study analyzed 839 eligible couples who had no missing values in the measurement variables (as described below, the number of individual “targets” included in our analyses was 1001).

The study protocol and informed consent were approved by the ethics committees of the Graduate School of Medicine of The University of Tokyo.

### Measurements

#### Smoking status

Respondents were asked to identify their smoking status from three categories (1 = current smoker, 2 = ex-smoker, 3 = never-smoker). Respondents who were categorized as current smokers or ex-smokers were then asked when they initiated smoking according to a year-month format. Ex-smokers were additionally asked when they quit smoking. In the spouse/partner survey, spouses were asked about their smoking status and the date of smoking initiation and cessation.

We extracted information about both spouses’ smoking status at marriage and their change in smoking status after marriage, based on wave 1 and spouse survey data. Then, we included all subjects who were smokers at the time of marriage as the targets. In couples who both smoked at the time of marriage, each person in the couple was counted in the analysis as a target, and their counterpart’s status at the time of behavioral change as an exposure. As a result, the number of individual targets in our analyses was 1001 (708 men and 293 women). Our main exposure variable was dichotomous, indicating whether the counterpart continued smoking after marriage or was a non-smoker (a never or ex-smoker at marriage, or quit after marriage). The outcome variable was also dichotomous, indicating whether the target respondent continued smoking after marriage or quit smoking during marital life (Table 1).

**Table 1:** **Combinations of outcomes and main exposure**

		Targets (outcome)	
		Quit after marriage	Continuing smoking
Counterparts (exposure)	Non-smoking (Ex-smoker at marriage/Quit after marriage/Never-smoker)	A	B
	Continuing smoking	C	D

#### Demographic variables

Educational attainment of high school graduation or lower was coded as 0 and that of college graduation or higher as 1. Educational attainment was measured as completion of the level of schooling at the time of the survey. In the analyses, combinations of the couple’s educational attainment were used as explanatory variables, i.e., we used four dummy variables indicating couples with a low-educated target and low-educated counterpart (LOW-low couple), a low-educated target and high-educated counterpart (LOW-high couple), a high-educated target and low-educated counterpart (HIGH-low couple), and a high-educated target and high-educated counterpart (HIGH-high couple). Additionally, the target respondent’s age and gender and household’s presence of children were also used as sociodemographic covariates.

### Statistical analysis

We adopted log-binominal regression models rather than logistic regression models as the prevalence of outcome events (change in smoking behavior after marriage) was relatively large [24, 25]. We set the target respondent’s change in smoking behavior as an outcome, and their counterpart’s non-smoking status as a main exposure.

First, we analyzed a model that included the main effect of counterpart’s non-smoking status and its interaction effect with the target’s sex to check whether there was a gender difference in the effect of counterpart’s smoking status. Because this analysis included some couples twice, the independence assumption of regression was violated. To account for potential underestimation of errors, we adopted robust error estimation to take within-couple clustering into consideration [26]. Because the interaction was significant, we further conducted analyses stratified by gender, by regressing the main effects of the counterpart’s non-smoking status and the couple’s educational attainment combinations on the target’s behavioral change in smoking after marriage (Model 1). In subsequent analyses, we added to Model 1 interaction terms between counterpart’s non-smoking status and couple’s educational attainment combinations to determine whether the spillover effect of counterpart’s non-smoking varied according to the couple’s educational levels (Model 2).

## Results

Table 2 presents descriptive statistics of the targets in the analysis.

**Table 2:** **Descriptive statistics of the target respondents**

	Men (n =708)		Women (n =293)	
	***n***	%	***n***	%
**Age**				
20-29	26	3.7	20	6.8
30-39	226	31.9	128	43.7
40-49	343	48.5	129	44.0
50 or older	113	16.0	16	5.5
**Presence of children (at least one more child)**				
Presence	587	82.9	243	82.9
Absence	121	17.1	50	17.1
**Couples’ educational combinations**				
Both spouses were low educated (LOW & low)	133	18.8	80	27.3
Target’s education was low and counterpart’s was high (LOW & high)	88	12.4	59	20.1
Target’s education was high and counterpart’s was low (HIGH & low)	88	12.4	38	13.0
Both spouses were high educated (HIGH & high)	399	56.4	116	39.6
**Target’s smoking status & Counterpart’s smoking status**				
Continued smoking & Continued smoking	113	16.0	112	38.2
Continued smoking & Non-smoking^a^	301	42.5	31	10.6
Quit smoking after marriage & Continued smoking	29	4.1	109	37.2
Quit smoking after marriage & Non-smoking^a^	265	37.4	41	14.0

^a^Counterpart’s “Non-smoking” includes “cessation before marriage,” “cessation after marriage but before target’ cessation,” and “never-smoked”.

About half of the target respondents were in their 40s, and about 80% had children; 31.2% of men and 47.4% of women achieved high school graduation or less. The percentage of the couples matched in terms of counterpart’s educational attainment (“LOW-low” or “HIGH-high” couples) were 75.2% among men and 66.9% among women. Whereas 42.5% of male targets continued smoking after marriage despite having a non-smoking counterpart, only 10.6% of female targets did so. Only 4.1% of males quit smoking despite their wife’s continuing smoking, whereas 37.2% of females did so. The proportion of couples in which both target and counterpart continued smoking after marriage was 16.0% among men and 38.2% among women.

Next, we examined whether the interaction term between the target’s sex and counterpart’s non-smoking was associated with the target’s cessation after marriage to check for a gender difference in the spillover effect (data not shown). The results showed that, in contrast to the non-significant main effect of counterpart’s non-smoking on the target’s postmarital cessation (risk ratio (RR) =1.20, 95% CI: 0.94–1.52), the interaction term was significant (RR =1.84, 95% CI: 1.22–2.76). A log-likelihood ratio (LR) test between the model with and without the interaction term showed that inclusion of the term was significant (LR chi^2^(1) =9.96, *p* < .01). Because these results indicated that there was a significant gender difference in the effect of counterpart’s non-smoking on the target’s cessation after marriage, the following analyses were stratified by gender.

Model 1 in Table 3 shows the log-binomial regression estimates for the effects of the counterpart’s non-smoking status and the couple’s educational attainment combinations on the target’s smoking cessation after marriage. Model 1 indicates that counterpart’s non-smoking was associated with the target’s smoking cessation after marriage among men (RR =2.27, 95% CI: 1.61–3.18), but not among women (RR =1.16, 95% CI: 0.90–1.49). The couple’s educational attainment combinations did not have significant main effects on smoking cessation in either men or women. As an additional analysis, we recategorized the counterpart’s non-smoking status into “never-smoker” and “ex-smoker” and examined whether these non-smoking categories showed different associations with the target’s cessation. The RRs of never-smoker counterparts were 2.04 for men and 1.02 for women whereas those of ex-smoker counterparts were 3.28 for men and 1.33 for women (continuing smoker as a reference, data not shown).

**Table 3:** **Log-binomial regression estimates of factors associated with target smoking cessation**

Outcome: target’s cessation after marriage	Men (n =708)		Women (n =293)	
Explanatory variables	Risk ratio	95% CI	Risk ratio	95% CI
**Model 1**				
Age				
20-29	0.85	(0.54-1.33)	1.10	(0.60-2.00)
30-39	0.65**	(0.51-0.82)	0.87	(0.54-1.39)
40-49	0.76**	(0.62-0.94)	0.96	(0.61-4.51)
50 or older	Reference		Reference	
Presence of children	1.07	(0.97-1.17)	1.12*	(1.01-1.24)
Couple’s educational combination^a^				
LOW & low couple	Reference		Reference	
LOW & high couple	0.84	(0.58-1.20)	0.84	(0.56-1.26)
HIGH & low couple	1.11	(0.81-1.53)	0.96	(0.64-1.43)
HIGH & high couple	1.02	(0.81-1.29)	1.25	(0.93-1.69)
Counterpart’s smoking status				
Continued smoking	Reference		Reference	
Quit smoking/Never smoking	2.27**	(1.61-3.18)	1.16	(0.90-1.49)
**Model 2**				
Age				
20-29	0.85	(0.54-1.33)	1.11	(0.59-2.10)
30-39	0.65**	(0.51-0.82)	0.87	(0.53-1.42)
40-49	0.76**	(0.62-0.93)	0.96	(0.59-1.55)
50 or older	Reference		Reference	
Presence of children	1.06	(0.97-1.16)	1.13*	(1.01-1.26)
Couple’s educational combination^a^				
LOW & low couple	Reference		Reference	
LOW & high couple	0.75	(0.23-2.43)	0.91	(0.58-1.42)
HIGH & low couple	1.08	(0.50-2.36)	0.99	(0.62-1.58)
HIGH & high couple	0.64	(0.26-1.57)	1.32	(0.94-1.85)
Counterpart’s smoking status				
Continued smoking	Reference		Reference	
Quit smoking/Never smoking	1.94*	(1.12-3.37)	1.55	(0.90-2.65)
Couple’s educational combination^a^ × counterpart’s smoking status				
LOW & low × counterpart’s non-smoking	Reference		Reference	
LOW & high × counterpart’s non-smoking	1.13	(0.33-3.89)	0.94	(0.51-1.74)
HIGH & low × counterpart’s non-smoking	1.03	(0.44-2.42)	1.16	(0.58-2.32)
HIGH & high × counterpart’s non-smoking	1.65	(0.65-4.20)	1.48*	(1.05-2.08)

Note: **p<.01, *p<.05.

^a^“LOW & low couple”: Both spouses had low education levels; “Low & high couple”: Target’s educationwas low and counterpart’s was high; “HIGH & low”: Target’s education was high and counterpart’s waslow; “HIGH & high couple”: Both spouses had high education levels.

Model 2 in Table 3 examined whether the association between a counterpart’s non-smoking and a target’s cessation varied depending on the couple’s educational combination. Targets in couples where both spouses had low levels of education (i.e., LOW-low couples) and the counterpart was a non-smoker were set to the reference category to examine whether the effect of a counterpart’s non-smoking depended on the couple’s educational combination. For men, any combination of educational attainment did not show significant interaction with counterpart’s non-smoking. However, for women, the interaction of high educational attainment in both spouses and their husband’s non-smoking showed a significant association with their own smoking cessation after marriage (RR =1.48, 95% CI: 1.05–2.08). This result suggested that women in couples where both spouses were highly educated and the husband was a non-smoker were 1.48 times more likely to stop smoking than women in couples where both had lower educational levels and the husband was a non-smoker. LR tests between models 1 and 2 demonstrated that interaction terms between educational pairs and spousal non-smoking as a whole were not significant either for men (LR chi^2^ = 1.39, *n.s.*) or for women (LR chi^2^ = 1.03, *n.s.*).

## Discussion

This study indicated that counterpart’s non-smoking had a major association with a target’s subsequent cessation in men, i.e., there was a spillover effect of the wife’s non-smoking only among men. However, husband’s non-smoking was not associated with female target’s cessation. In women, a significant association between counterpart’s non-smoking and the target’s own cessation was only observed in couples in which both spouses were highly educated. The results suggested that the spillover effect from highly educated husbands quitting tobacco use was effective only for highly educated wives. However, among men, the combinations of the couples’ educational levels had no influence on the spillover effect. The strength of the present study is that the possibility of assortative mating was excluded to some extent by analyzing the target respondents’ postmarital behavioral changes in smoking.

Intrinsically, one person in a partnership may regulate the partner’s health behaviors through direct physical intervention in an effort to improve the health of their partner [27]. Although many spouses generally monitor and attempt to control their partner’s health behaviors, women are more likely to attempt to control their spouse’s health than men [16]. Our finding that men benefit from their female counterpart’s non-smoking, regardless of her educational background could be explained by the general influence of women on the daily habits of their husbands by monitoring health and social behavior, and/or providing support for behavioral change. Meanwhile, our analyses suggested that a husband’s non-smoking was significant only for women in highly educated couples, suggesting that a couple’s educational level may modify the impact of a husband’s non-smoking on women. One plausible reason is that the amount of social influence and/or support that husbands exert on their wives depends on their educational backgrounds. A woman who marries a husband of low educational attainment may receive relatively little social support from him to stop smoking (e.g., emotional encouragement), or may have a negative influence from him (e.g., emotional pressure to smoke) [28]. Likewise, a woman’s tendency to accept a husband’s influence may also depend on her own educational level. For example, a high education increases knowledge about health and helps people accept the positive influences from family health behaviors.

The analyses that split never-smoker and ex-smoker counterparts into independent categories indicated that the spillover effect for a male target from his counterpart may depend on whether the counterpart was a never-smoker or ex-smoker, and this was consistent with the findings of Monden et al. [10]. Ex-smoker counterparts may be more likely to dislike their spouse smoking and to intervene in their spouse’s cessation than never-smoker counterparts, or ex-smoker counterparts may be able to provide more appropriate support for their smoking spouse because they know the difficulty of quitting smoking.

One possible reason that the main effect of a couple’s educational level was not significant in this study may be attributed to the failure of previous studies to exclude the possibility of assortative mating [17, 18]. That is, there is a possibility that a highly educated person marries a highly educated partner and, in such couples, the probability of being a non-smoker is high because of their high educational level. In fact, when examining the cross-sectional smoking status in our data, it was shown that the target’s own and the counterpart’s educational levels were associated with the target’s smoking status. In this paper, we focused on smoking cessation after marriage and treated the respondent who was a smoker at the start of their marital life as the target, thus it is reasonable to believe that the main effect of educational attainment is weakened in our analyses, with a reduced influence of assortative mating.

Several limitations should be noted in this study. First, we measured the respondents’ self-reported date of smoking cessation, which may be susceptible to measurement errors. Second, although partners who were living with, but were not legally married to the respondents in the wave 1 survey (e.g., common-law husband/wife) were also invited to participate in the spouse/partner survey, they were omitted from our analyses because of the lack of a defined starting date of the partner relationship. However, an unmarried partner can also be seen as influential in the issue of the spillover effect from intimate others. Future research should include unmarried couples and examine the effect of the intimate partner’s smoking behavior. Third, the sample was derived from four municipalities in the Greater Tokyo metropolitan area, which may affect the generalizability of our findings. Finally, we simply assumed in our analysis that the initial cessation of a spouse affected the subsequent cessation of the partner; however, temporal initiation does not necessarily signify a causal association between the behaviors, and our estimates of the spillover effect may have been exaggerated.

The implications of our results for public health practice are that smoking cessation programs targeting both spouses may be more effective than those targeting individuals in some couples. For example, for couples who both smoke, if either one can successfully quit smoking, the eventual likelihood of success of both spouses quitting can be increased. Or, if a practitioner finds that one spouse’s likelihood of cessation is higher than that of the other spouses’, initial intervention for the former spouse can increase the probability of cessation in both spouses. Thus, both spouses should be involved in the intervention. This is especially the case for men. However, for women, the husband’s smoking status may not enhance the effectiveness of the intervention if the educational attainment of both spouses is not high; our result implied that the spillover effect between spouses may not be strong in such couples.

## Conclusion

Our findings suggest that a spouse's smoking status is associated with men's smoking cessation. For women, however, a couple's combined educational attainment may matter in that association. The present paper implies that cessation programs should involve both members of a couple, and such programs should take into account the educational backgrounds of the couple in the case of women smokers.
